# Sensitivity of joint contagiousness and susceptibility-based dynamic optimal control strategies for HIV prevention

**DOI:** 10.1371/journal.pone.0204741

**Published:** 2018-10-18

**Authors:** Ingo Bulla, Ian H. Spickanll, Dmitry Gromov, Ethan Obie Romero-Severson

**Affiliations:** 1 Department of Mathematics and Computer Science, University of Greifswald, Greifswald, Germany; 2 Division of STD Prevention, Centers for Disease Control and Prevention, Atlanta, Georgia, United States of America; 3 Faculty of Applied Mathematics and Control Processes, Saint Petersburg State University, Saint Petersburg, Russia; 4 Theoretical Biology and Biophysics Group, Los Alamos National Laboratory, Los Alamos, New Mexico, United States of America; Jiangsu provincial Center for Disease Control and Prevention, CHINA

## Abstract

Predicting the population-level effects of an infectious disease intervention that incorporate multiple modes of intervention is complicated by the joint non-linear dynamics of both infection transmission and the intervention itself. In this paper, we consider the sensitivity of Dynamic Optimal Control Profiles (DOCPs) for the optimal joint investment in both a contagiousness and susceptibility-based control of HIV to bio-behavioral, economic, and programmatic assumptions. The DOCP is calculated using recently developed numerical algorithms that allow controls to be represented by a set of piecewise constant functions that maintain a constant yearly budget. Our transmission model assumes multiple stages of HIV infection corresponding to acute and chronic infection and both within- and between-individual behavioral heterogeneity. We parameterize a baseline scenario from a longitudinal study of sexual behavior in MSM and consider sensitivity of the DOCPs to deviations from that baseline scenario. In the baseline scenario, the primary determinant of the dominant control were programmatic factors, regardless of budget. In sensitivity analyses, the qualitative aspects of the optimal control policy were often robust to significant deviation in assumptions regarding transmission dynamics. In addition, we found several conditions in which long-term joint investment in both interventions was optimal. Our results suggest that modeling in the service of decision support for intervention design can improve population-level effects of a limited set of economic resources. We found that economic and programmatic factors were as important as the inherent transmission dynamics in determining population-level intervention effects. Given our finding that the DOCPs were robust to alternative biological and behavioral assumptions it may be possible to identify DOCPs even when the data are not sufficient to identify a transmission model.

## Introduction

Infectious disease intervention effects play out across multiple scales: they protect a person from transmitting or becoming infected at the individual-level, which has population-level effects by decreasing the overall force of infection as a result of these individual-effects (i.e., herd immunity). Predicting the population-level effectiveness of an intervention is difficult because population-level effects 1) are heterogeneous between populations, and 2) are heterogeneous within populations over time. While individual-level effects can be measured in appropriate animal models and carefully designed trials [1,2], and subsequent population-level effects can be predicted using mathematical modeling, intervention feasibility as it relates to costs and logistics is also important to consider. For example, a hypothetical Human Immunodeficiency Virus (HIV) drug with 100% efficacy at clearing infection from an individual may not be feasible due to prohibitive cost or availability. The joint non-linear dynamics of both transmission and intervention coupled to budgetary and programmatic factors produce complex patters of intervention effectiveness both within and between populations.

Discovering optimal mixtures of intervention strategies has been identified as a key aspect of the program science paradigm for public health intervention [3,4]. As interventions are rolled out, they are funded at specific levels, thereby determining intervention coverage after accounting for intervention costs. Given two interventions, the relative funding level of each intervention (and therefore the resulting levels of each intervention’s coverage) defines a specific control policy. When the control policy can change each year, the multi-year control policy is defined as the dynamic control policy. There are a very large number of potential dynamic control policies over a multi-year period, which makes finding optimal control policies challenging. We recently developed a computational framework for computing Dynamic Optimal Control Policies (DOCPs) [5] that are defined as the year-to-year relative investment in alternative prevention strategies that minimize the number of incident cases over a specific period. Our method makes several important advances in numerical optimal control that we apply in this paper. First, we represent controls as year-to-year step-functions representing funding of a specific intervention, which allows the control to be flexible in response to changing dynamics. We also consider the joint space of the controls over the entire period of interest. For example, when we calculate the DOCP for 2 controls over a 50-year time frame, we optimize over the full 100-dimentional space (1 parameter for each control for each year). This allows us to take a very long view of optimal control while still accounting for both the effect of the control on the system dynamics and the possibility of external changes to the system. We show in this paper that our method can identify long-term trade-offs in different controls that would be impossible to identify in short-term analyses. Second, we model the costs of not only enrolling eligible individuals but also the cost of attempting to enroll people who are ineligible. By doing so, we naturally penalize interventions that are dependent on enrolling hard to identify people. Finally, our method obeys a strict, and not necessarily constant, budget in each year.

The methods we have developed and employed in this paper are based on the mathematics of control theory that has been used to address epidemiological resource allocation problems since the 1970s [6]. However, much of the research applying control theory to epidemiologic problems has focused on purely theoretical concerns [7–9], calculated the optimal allocation strategy for very general settings [10–16], or assumed a strong a priori form to the control [17]. Studies where the underlying model and choice of parameters were tailored to a specific setting [18,19] were less frequent than general studies. Our long-term goal is to provide a pragmatic and rationale bridge between control theoretic methods and infectious disease policy.

We applied our method to a mathematical model of HIV transmission in a hypothetical population of men who have sex with men (MSM) considering deviations from a baseline model along five axes representing different aspects of transmission dynamics. We consider variable budget levels and alternative policy restrictions. We consider two interventions which we refer to as controls: a contagiousness control parameterized to have costs similar to a Treatment-as-Prevention (TasP) approach and a susceptibility control parameterized to have costs similar to a Pre-Exposure Prophylaxis (PrEP).We found that qualitative aspects of DOCPs are often robust to a range of modeling assumptions and that the optimal allocation of resources is often determined by assumptions about the deployment of the intervention rather than the biological and behavioral properties of the transmission system alone.

## Materials and methods

### Nomenclature

We use the terms *study period*, *analytic horizon*, and *step size* to reflect specific concepts defined here for clarity. We define study period as the period of time over which the DOCP will be reported. We define analytic horizon as the period over which the DOCP will be calculated. The analytic horizon must by definition be longer than or equal to the study period. When the analytic horizon is not much longer than the study period, artifacts may arise as the algorithm becomes “shortsighted”. For example, if one control is very effective in the short-term but ineffective in the long-term, it might be preferred as the end of the analytic horizon is approached. This artifact can be avoided by having an analytic period much longer than the study period. We define step size as the time between steps in the step function; the control policy is constant within a given step.

In the analyses that we report in this paper, the study period is 50 years, the analytic horizon is 70 years, and the step size is 1 year. We selected the analytic horizon such that we believe it is unlikely that we have any artifacts caused by shortsightedness; at no point is the algorithm calculating the DOCP without looking at least 20 years into the future. We selected a study period of one year to balance the temporal resolution of the control with the basic reality that policies cannot be changed instantaneously.

### Transmission model

**Fig 1:**
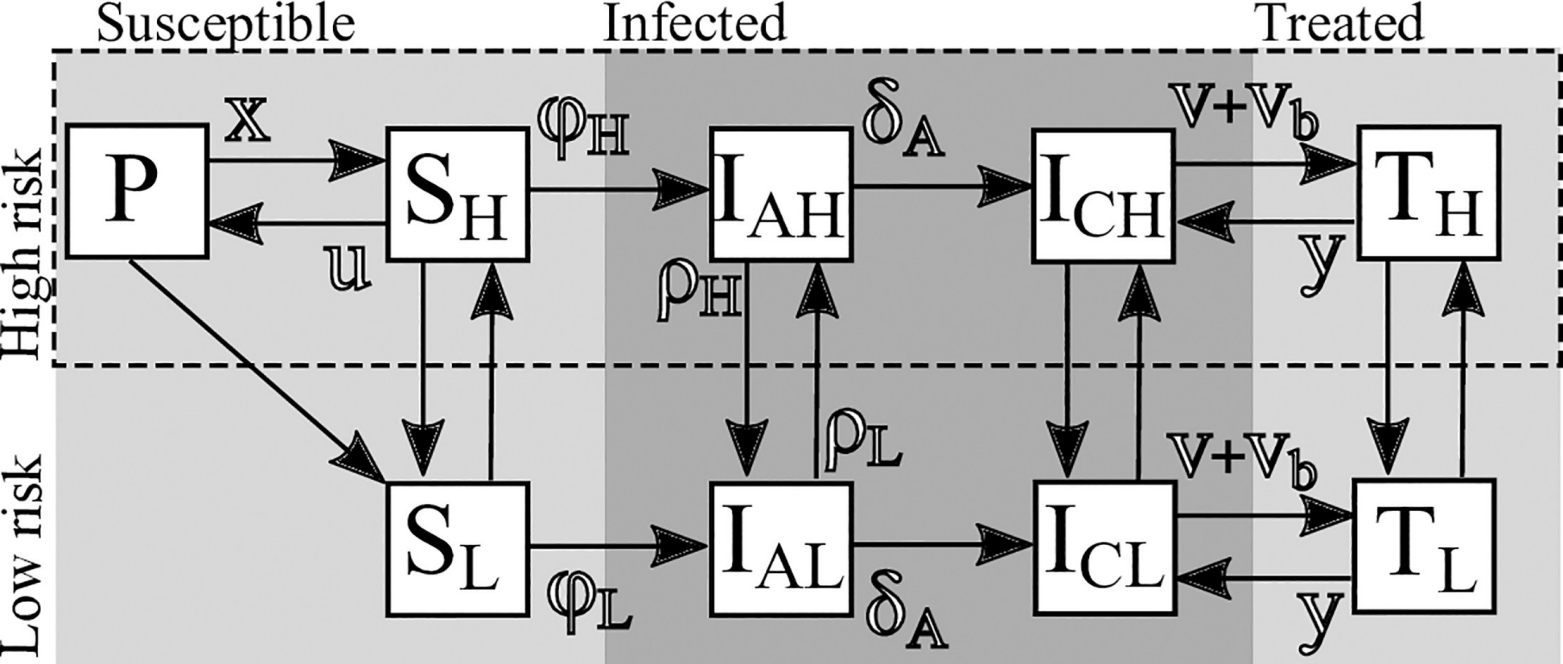
Illustration of the transmission model. The transmission model divides the population into 9 states (Treated High Risk, Treated Low Risk, Chronic High Risk, Chronic Low Risk, Actue High Risk, Acute Low Risk, Susceptible High Risk, Susceptible Low Risk, and Protected) represented by boxes. Flows between states are represented as arrows. Symbols represent rate coefficients and model parameters. The term *ϕ*_*H*_ and *ϕ*_*L*_ are complex terms involving both sexual mixing and contact rate terms.

We represented our model of MSM HIV transmission dynamics (Fig 1 and Table 1) as a set of ordinary differential equations encompassing 9 states dividing individuals between susceptible (S), infected (I), virally suppressed due to treatment (T), and protected susceptible due to susceptibility intervention (P). Individuals can also be high or low-risk (H and L subscripts respectively) and infected individuals can be acutely or chronically infected (A and C subscripts respectively). We indicate the risk-status as a subscript and state as a capital letter (e.g., *I*_*AH*_ indicates the set of acutely infected, high-risk individuals). Mixing is assumed to follow the “preferred” formulation from Jacquez et al^22^. This general model structure has been used to study the interaction of acute-stage contagiousness, behavior variability, and the efficacy of TasP as a public health intervention [5,22,23]. The system of equations is

$$\dot{S_{H}}=\alpha_{H}-(\phi_{H}+\rho_{H}+\mu )S_{H}+\rho_{L}S_{L}+xP-u\zeta_{P}N$$

$$\dot{S_{L}}=\alpha_{L}-\left(\phi_{L}+\rho_{L}+\mu \right)S_{L}+\rho_{H}\left(S_{H}+P\right)$$

$$\dot{I_{CH}}=\delta_{A}I_{AH}-(\rho_{H}+\mu +\delta_{C}+v_{b})I_{CH}+\rho_{L}I_{CL}+yT_{H}-v\zeta_{TH}N$$

$$\dot{I_{CL}}=\delta_{A}I_{AL}-(\rho_{L}+\mu +\delta_{C}+v_{b})I_{CL}+\rho_{H}I_{CH}+yT_{L}-v\zeta_{TL}N$$

$$\dot{I_{AH}}=\phi_{H}S_{H}-(\rho_{H}+\mu +\delta_{A})I_{AH}+\rho_{L}I_{AL}$$

$$\dot{I_{AL}}=\phi_{L}S_{L}-(\rho_{L}+\mu +\delta_{A})I_{AL}+\rho_{H}I_{AH}$$

$$\dot{T_{H}}=-(y+\rho_{H}+\mu )T_{H}+v_{b}I_{CH}+\rho_{L}T_{L}+u\zeta_{TH}N$$

$$\dot{T_{L}}=-(y+\rho_{L}+\mu )T_{L}+v_{b}I_{CL}+\rho_{H}T_{H}+u\zeta_{TL}N$$

$$\dot{P}=-(x+\rho_{H}+\mu )P+v\zeta_{P}N$$

where *ϕ*_*H*_ and *ϕ*_*L*_ are the per-capita infection rate for high and low-risk susceptibles respectively. To derive these quantities, we consider the total number of contacts made at a given time

$$\theta =\lambda_{H}N_{H}+\lambda_{L}N_{L}$$

where

$$N_{H}=S_{H}+I_{CH}+I_{AH}+T_{H}+P$$

$$N_{L}=S_{L}+I_{CL}+I_{AL}+T_{L}$$

$$N=N_{H}+N_{L}.$$

Then, the probability that a given random individual has a contact with a high and low-risk individual at the common mixing site is

$$\eta_{H}=\frac{\lambda_{H}N_{H}}{\theta },\eta_{L}=\frac{\lambda_{L}N_{L}}{\theta }$$

respectively. Then, the probability of a transmission given a contact at the common site is

$$\sigma =\beta_{A}\left(\eta_{H}\frac{I_{AH}}{N_{H}}+\eta_{H}\frac{I_{AL}}{N_{L}}\right)+\beta_{C}\left(\eta_{H}\frac{I_{CH}}{N_{H}}+\eta_{L}\frac{I_{CL}}{N_{L}}\right).$$

Therefore, the per-capita rate of a high-risk susceptible becoming infected at the common site is

$$\tau_{H}=(1-\pi )\lambda_{H}\sigma .$$

At the preferred mixing site the per-capita transmission rate for high-risk susceptibles becoming infected is

$$\psi_{H}=\pi \lambda_{H}\left(\beta_{A}\frac{I_{AH}}{N_{L}}+\beta_{C}\frac{I_{CH}}{N_{H}}\right).$$

Therefore *ϕ*_*H*_ = *τ*_*H*_ + *ψ*_*H*_. The derivation of *ϕ*_*L*_ proceeds in the same manor but with subscripts corresponding to the low-risk state variables.

**Table 1:** Transmission model states and parameters.

Parameter/State	Description	Value	Ref
*S*_*H*_	Number of high-risk susceptibles	state variable	
*S*_*L*_	Number of low-risk susceptibles	state variable	
*I*_*AH*_	Number of high-risk acute infecteds	state variable	
*I*_*AL*_	Number of low-risk acute infecteds	state variable	
*I*_*CH*_	Number of high-risk chronic infecteds	state variable	
*I*_*CL*_	Number of low-risk chronic infecteds	state variable	
*T*_*H*_	Number of high-risk treated persons	state variable	
*T*_*L*_	Number of low-risk treated persons	state variable	
*P*	Number of people on PrEP	state variable	
*α*_*H*_	Entry rate into high-risk category	250	
*α*_*L*_	Entry rate into low-risk category	28	
*λ*_*H*_	High-risk contract rate	7.92 ∙ *λ*_*L*_	[20]
*λ*_*L*_	Low-risk contract rate	fit	
*β*_*A*_	Acute probability of transmission	varied	[21]
*β*_*C*_	Chronic probability of transmission	0.01	assumption
*ρ*_*H*_	Rate from high to low-risk state	*ρ*_*L*_ ∙ 9^−1^	assumption
*ρ*_*L*_	Rate from low to high-risk state	varied	
*π*	Proportion of contacts made at risk-specific site	varied	
*δ*_*A*_	Progression from acute to chronic stage	4^−1^	assumption
*δ*_*C*_	Progression from chronic-stage to death	116^−1^	assumption
*μ*	Removal rate	(30 ∙ 12)^−1^	assumption
*ζ*_*G*_	Probability of sampling someone in state G	derived	see below
*ϕ*_*H*_	Force of infection for high-risk susceptibles	derived	see below
*ϕ*_*L*_	Force of infection for low-risk susceptibles	derived	see below
*v*_*b*_	Baseline rate of treatment	fit	
*x*	Rate PrEP is stopped	varied	assumption
*y*	Rate TasP is stopped	0	assumption
*u*	PrEP control rate	fit	
*v*	TasP control rate	fit	

The model parameters can be interpreted in the following way: *δ*_*A*_ is the rate that acutely infected persons become chronic infected, *δ*_*C*_ is the rate that chronically infected persons are removed due to AIDS, *β*_*A*_ is the per-act probability of transmission with an acute infected person, *β*_*C*_ is the per-act probability of transmission with a chronic infected person, *π* is the probability that a contact occurs at the preferred mixing site, *λ*_*H*_ is the contact rate of high-risk persons, *λ*_*L*_ is the contact rate of low-risk persons, x is the rate at which the susceptibility control fails, y is the rate at which the infectiousness control fails, *μ* is the general removal rate, *ρ*_*H*_ is the rate that high-risk persons become low-risk, and *ρ*_*L*_ is the rate that low-risk persons become high-risk.

### Intervention model eligibility and duration

We model two interventions that have either contagiousness or susceptibility effects. The contagiousness intervention costs are parameterized to emulate Treatment-as-Prevention (TasP) and the susceptibility intervention costs are parameterized to emulate Pre-Exposure Prophylaxis (PrEP). We assumed that intervention enrollment occurs at a venue that is enriched for high-risk individuals (4-fold increased probability of finding high-risk individuals by random chance compared to the general population). Individuals are selected at random and tested for HIV infection. High-risk susceptibles are eligible for PrEP and infected but unware individuals are eligible for TasP. The intervention model accounts for both the probability distribution of states in the high-risk venue and the differential costs of attempting to enroll different people in each intervention. We assumed that all at-risk persons are potentially enrollable, no one refuses to be tested or treated, and individuals are fully compliant once enrolled. We considered two durations of PrEP administration: either 1 year (“1-Year PrEP”) or for the duration of the high-risk period (“Unlimited PrEP”). At the end of a high-risk interval we assumed that PrEP is discontinued.

### Intervention model eligibility and duration

We model two interventions that have either contagiousness or susceptibility effects. The contagiousness intervention costs are parameterized to emulate Treatment-as-Prevention (TasP) and the susceptibility intervention costs are parameterized to emulate Pre-Exposure Prophylaxis (PrEP). We assumed that intervention enrollment occurs at a venue that is enriched for high-risk individuals (4-fold increased probability of finding high-risk individuals by random chance compared to the general population). Individuals are selected at random and tested for HIV infection. High-risk susceptibles are eligible for PrEP and infected but unware individuals are eligible for TasP. The intervention model accounts for both the probability distribution of states in the high-risk venue and the differential costs of attempting to enroll different people in each intervention. We assumed that all at-risk persons are potentially enrollable, no one refuses to be tested or treated, and individuals are fully compliant once enrolled. We considered two durations of PrEP administration: either 1 year (“1-Year PrEP”) or for the duration of the high-risk period (“Unlimited PrEP”). At the end of a high-risk interval we assumed that PrEP is discontinued.

The cost function takes the form

$$\sum_{G\in \Gamma }k_{T}^{\left(e\right)}(G)P(G|HRE)N(t)v(t)+\sum_{G\in \Gamma }k_{P}^{\left(e\right)}(G)P(G|HRE)N(t)u(t),$$

where Γ = {S_H_,S_L_,A_H_,A_L_,C_H_,C_L_,T_H_,T_L_,P} is the set of all possible states that a potential enrollee can be in. This equation states that the per-person cost for attempting to enroll *N*(*t*)*v*(*t*) and *N*(*t*)*u*(*t*) people into TasP and PrEP, respectively, is the per-person cost for that category of person for the TasP and PrEP interventions, $k_{T}^{\left(e\right)}(G)$ and $k_{P}^{\left(e\right)}(G)$, respectively, times the probability of finding that type of person at the intervention venue, *P*(*G*|*HRE*)—this term is called *ζ*_*G*_ for simplicity in the model equations; the term HRE simply refers to the fact that the intervention is assumed to be taking place in a High-Risk Environment. Because we assume that the intervention attempts to enroll people at random, we can define the term *P*(*G*|*HRE*) as the relative frequency of each type of person in the high-risk environment. The final relevant term is the odds ratio of finding a high-risk person in the high-risk environment, which we refer to as *r*_*b*_. Splitting the set of states into the high-risk set, Γ_H_ = {S_H_,A_H_,C_H_,T_H_,P}, and the low-risk set, Γ_L_ = {S_L_,A_L_,C_L_,T_L_}, we can now write down an explicit form for *P*(*G*|*HRE*):

$$P(G_{H}|HRE)=\frac{P(HRE|G_{H})P(G_{H})}{P(HRE)}=\frac{p_{H}\frac{G_{H}}{N}}{p_{H}\frac{N_{H}}{N}+p_{L}\frac{N_{L}}{N}}=\frac{r_{b}G_{H}}{r_{b}N_{H}+N_{L}},\;\forall G_{H}\in \Gamma_{H},$$

and

$$P(G_{L}|HRE)=\frac{P(HRE|G_{L})P(G_{L})}{P(HRE)}=\frac{p_{L}\frac{G_{L}}{N}}{p_{H}\frac{N_{H}}{N}+p_{L}\frac{N_{L}}{N}}=\frac{G_{L}}{r_{b}N_{H}+N_{L}},\;\forall G_{L}\in \Gamma_{L}.$$

Finally, to define the cost of attempting to enroll someone we need to define the 18 possible combinations of the 2 interventions and the 9 possible states that a person can be in. To account for each possibility we break each case down into the associated costs for each encounter as a sum of the structural costs and the specific costs of laboratory tests for each case. We assume that there is a general structural cost of setting up the interview that applies to all encounters, A = 267 [24], and specific costs associated with a positive rapid test, L1P = 92 [25]; a negative rapid test, L1N = 21[25]; a positive immunoassay, L2P = 69[25]; and a negative immunoassay, L2N = 10 [25]. Table 2 gives the values of the per enrollment costs and the assumptions that we used in obtaining those numbers. All costs were adjusted to 2010 USD values; the structural costs were assumed to follow general inflation while the medical costs increased by the medical inflation index.

**Table 2:** Cost function parameters. Includes the following costs, structural cost of an interview A = 267, positive rapid test L1P = 92, negative rapid test L1N = 21, positive immunoassay L2P = 69, and negative immunoassay L2N = 10.

Target, G	PrEP cost – $k_{P}^{\left(e\right)}(G)$	TasP cost – $k_{T}^{\left(e\right)}(G)$
SH	A+L1N+L2N (369)	A+L1N (288)
SL	A (267)	A+L1N (288)
AH	A+L1P (359)	A+L1P+L2P (482)
AL	A+L1P (359)	A+L1P+L2P (482)
CH	A+L1P (359)	A+L1P+L2P (482)
CL	A+L1P (359)	A+L1P+L2P (482)
TH	A (267)	A (267)
TL	A (267)	A (267)
P	A (267)	A (267)

Integrating in the costs of ongoing treatment, we have the final cost function in terms of the TasP and PrEP controls

$$V(v,u)=\int_{0}^{T}k_{T}(T_{H}(t)+T_{L}(t))+k_{P}P(t)+\sum_{G\in \Gamma }k_{T}^{\left(e\right)}(G)P(G|HRE){N(t)u}_{T}(t)+\sum_{G\in \Gamma }k_{P}^{\left(e\right)}(G){P(G|HRE)N(t)u}_{P}(t)\;dt$$

where *k*_*P*_ = 846 is the monthly cost of PrEP [26] and *k*_*T*_ = 1942 is the monthly cost of TasP [27].

### Transmission model parameterization

The baseline model was parameterized according to previous work [20]. In that paper contact rates were found to be Gamma distributed with mean 1.75 and standard deviation 2.67. We assumed that 10% of the infection-free population is high-risk and therefore fixed the ratio of contact rates by

$$\frac{\lambda_{H}}{\lambda_{L}}=\frac{\int_{4.88}^{\infty }f(z)zdz}{\int_{0}^{4.88}f(z)zdz}=7.92$$

where *f* is the density of contact rates and 4.88 is the 90^th^ percentile. The same study found behavioral intervals lasted on average for 2 years. Therefore, we set $\rho_{H}=\frac{1}{24}$ and $\rho_{L}=\frac{1}{216}$ giving a mean duration of 2 and 18 years for high and low-risk periods. The per act probability of transmission in the chronic stage was assumed to be 0.001 and the ratio of acute to chronic-stage contagiousness of 15 [21]. To obtain a full parameter set, the values of *λ*_*L*_ and *v*_*b*_ were selected to give 20% endemic prevalence [28] and 25% viral suppression [29] for each of the considered parameter sets.

We defined parameter sets to represent “high” and “low” values—with the baseline parameterization (Table 3) being the “center” value—along 5 axes representing modeling aspects that are either generally unknown or might be variable between populations.

**Table 3:** Parameter values.

	*λ*_*L*_	*λ*_*H*_	*β*_*A*_	*β*_*C*_	*μ*	*δ*_*A*_	*δ*_*C*_	*ρ*_*H*_	*ρ*_*L*_	*π*	*α*	*v*_*b*_	*R*_0_	*τ*_*A*_
Static Behavior	5.5	43.6	0.015	0.001	1/360	1/4	1/116	0	0	0.5	278	0.00097	4.5	0.43
**Baseline**	**3.2**	**25.2**	**0.015**	**0.001**	**1/360**	**1/4**	**1/116**	**1/24**	**1/216**	**0.5**	**278**	**0.00097**	**1.5**	**0.58**
Volatile Behavior	3.6	28.7	0.015	0.001	1/360	1/4	1/116	1/6	1/54	0.5	278	0.00097	1.3	0.59
Low Acute Contag.	5.3	42.0	0.005	0.001	1/360	1/4	1/116	1/24	1/216	0.5	278	0.00097	1.5	0.31
High Acute Contag.	1.3	10.4	0.05	0.001	1/360	1/4	1/116	1/24	1/216	0.5	278	0.00097	1.6	0.82
Random Mixing	3.0	24.1	0.015	0.001	1/360	1/4	1/116	1/24	1/216	1	278	0.00097	1.8	0.60
Self-Only Mixing	3.5	27.5	0.015	0.001	1/360	1/4	1/116	1/24	1/216	0	278	0.00097	1.4	0.56
Low Prevalence	2.1	16.9	0.015	0.001	1/360	1/4	1/116	1/24	1/216	0.5	278	0.00097	1.02	0.60
High Prevalence	5.5	43.5	0.015	0.001	1/360	1/4	1/116	1/24	1/216	0.5	278	0.00097	2.7	0.55
Low Suppression	3.2	25.6	0.015	0.001	1/360	1/4	1/116	1/24	1/216	0.5	278	0	1.6	0.56
High Suppression	3.2	25.2	0.015	0.001	1/360	1/4	1/116	1/24	1/216	0.5	278	0.00294	1.5	0.61

The first set, “behavior”, allows for a variable frequency of high-risk episodes that a person will experience in their lifetime, which is known to be a strong determinant of HIV transmission dynamics [22,23,30]. The “static” case assumed that risk-behavior is constant while the “volatile” case assumed that switching between high and low-risk phases was faster than in the baseline model. The static case might correspond to a population where a stable subset of the population has some high-risk behavior (e.g. highly sexually active people), while the volatile case might correspond to a population where individuals engage in periodic high-risk behavior (e.g. occasional partying). Sexual behavior is especially important because it is likely one of the main theoretical differences between populations with respect to HIV transmission and also because it can be used as a criterion for eligibility for certain interventions. For example, the CDC guidelines for PrEP direct clinicians to discuss various risk behaviors when considering PrEP for eligible patients [31].

The second set, “acute contagiousness”, allows for the difference in the relative contagiousness of the initial acute stage of infection, which is both difficult to estimate and contentious [21,32]. This factor matters because TasP effectiveness ought to decrease if people are diagnosed only in the chronic phase (i.e. TasP cannot prevent infections from acutely infected person) with increasing acute-stage contagiousness. Acute-stage contagiousness is also likely to be variable between populations due to biological synergy from the coincidence of other STIs [33].

The third factor is “mixing”, which governs how frequently different infectious types contact one another, is generally very difficult to measure directly but is also known to be a determinant of transmission dynamics and extinction criteria [34]. However, new phylogenetic methods have proven useful for potentially identifying sexual mixing patterns within populations [35,36]. In “random mixing” individuals pick partners from the general population at random (i.e. with no specific preference for their partners risk behavior) while in “self-only mixing” high-risk person only mix with other high-risk persons and the same for low-risk persons.

The last two axes consider different levels of prevalence and viral suppression at the start of the intervention. These factors represent the most basic ways that HIV epidemics are different between populations. The “low” levels are 1% and 0% for prevalence and viral suppression respectively while the “high” levels are 50% for both prevalence and viral suppression. The parameters for all scenarios are given in Table 3 (units are per month).

We assume that once viral suppression is obtained that persons remain suppressed (*y* = 0), and that the rate at which PrEP is stopped is $x=\frac{1}{12}$ in the “1-year PrEP” parameter set and *x* = 0 in the “Unlimited PrEP” case. That is we assume that persons are fully compliant with both TasP and PrEP and that PrEP ends only when a high-risk interval ends or PrEP runs out.

### Outcomes

As outcomes, we have summarized the DOCP for each scenario as the number enrolled into each intervention program from year to year; that is, we plot the integral of the flow from the target states to the protected or treated states due to the intervention rather, which is more interpretable than the raw control. We have also illustrated the annual proportional reduction in HIV incidence that results from the DOCP compared to no intervention, as well the reduction that occurs when only a single control policy is funded for the entire course. Note that in both the single-control and DOCP cases, the optimal control that reduces the cumulative incidence over the analytic horizon is computed. Besides cumulative incidence, we have also calculated the approximate proportion of transmissions from acutely infected individuals pre-intervention, *τ*_*A*_, and the basic reproduction number, *R*_0_, for each parameter set according to the next-generation matrix method [37] (Table 3).

### Numerical methods for finding dynamic optimal control policies

We previously published numerical algorithms for finding DOCPs using this model as an example [5]. Briefly, the optimal control policies were computed by using the orthogonal collocation method. This approach consists of parametrizing both the controls and the system trajectory and then determining the missing values of parameters by solving a large system of nonlinear algebraic equations. Along with system’s equations, the constraints are described by using the introduced parametrization. The optimal control problem is thus formulated as a large-scale nonlinear optimization problem which is solved using a sequential quadratic programming (SQP) solver. Code implementing these algorithms was written in Matlab [38] and was used to find DOCPs for this study. Example analysis code is included as a supplement.

## Results

**Fig 2:**
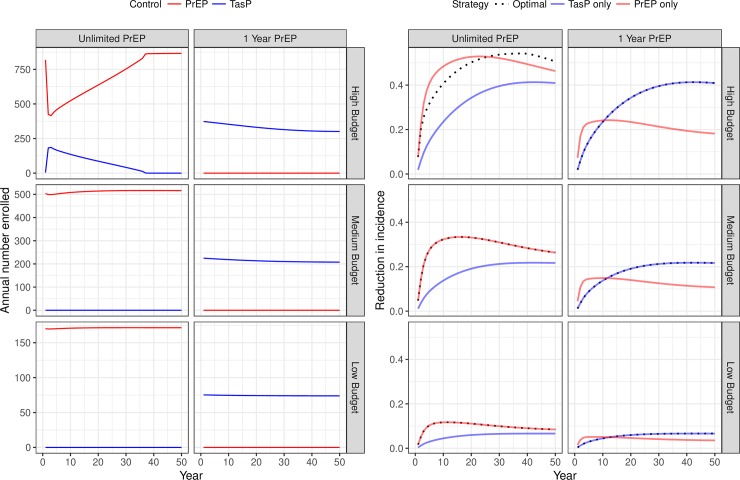
Dynamic optimal control policies and intervention effects in the baseline scenario. The left panel gives the optimal number of individuals annually enrolled in TasP (red) and PrEP (blue) for 1, 3, and 5 million dollars per year (low, medium, and high budgets respectively) and two different PrEP policies, “Unlimited PrEP” for PrEP provided for the entire duration of a high-risk period or “1-Year PrEP” for PrEP provided for 1 year. The right panel gives the multiplicative-scale reduction in annual incidence for the dynamic optimal (black dots), PrEP-only (blue), and TasP-only (red) control policies.

The allocation of limited resources between PrEP and TasP produces large differences in population effectiveness. Fig 2 (left) shows the DOCP for the baseline scenario as a function of both the annual budget and the duration of PrEP. Fig 2 (right) shows the proportional reduction in incidence in the baseline scenario under three dynamic control policies: the DOCP, TasP-only, and PrEP-only. The difference between the allocations that we considered (the DOCP, TasP-only, and PrEP-only) were generally large. For example, in the high budget scenario (5 million dollars per year) the difference in the optimal policies in terms of the number of cases prevented over the 50-year study period was 11,201 and 8,596 in the unlimited PrEP and 1-year PrEP scenarios respectively. However, when the DOCP was heterogeneous (i.e. involving both PrEP and TasP) the difference between the DOCP and the next best optimal homogenous control was generally much smaller. For example, the heterogeneous DOCP in the unlimited-PrEP, high-budget, scenario prevents only 562 more cases over the 50-year period than the only-PrEP allocation.

**Fig 3:**
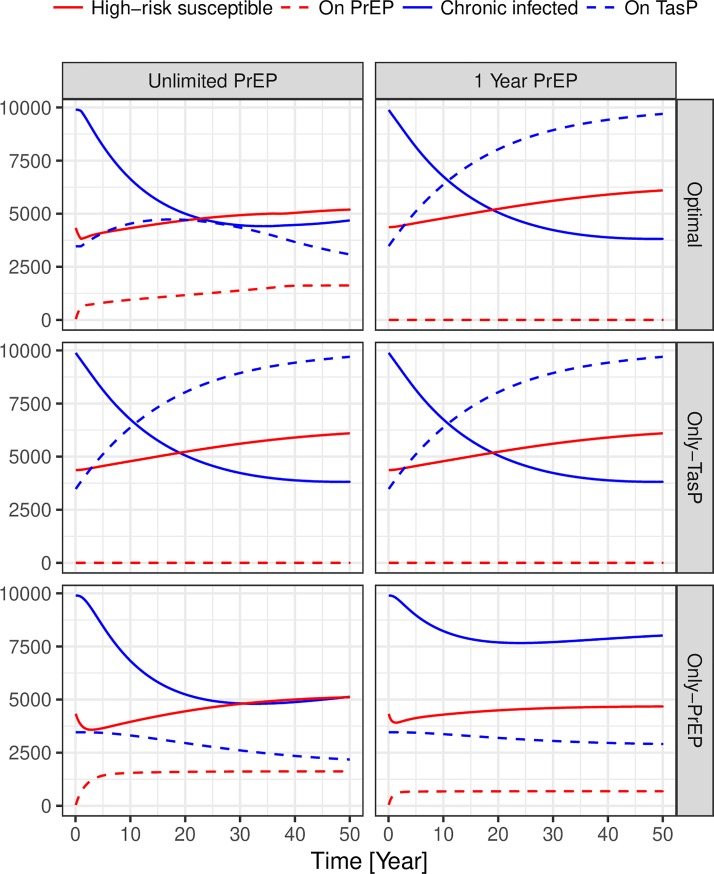
Number of possible enrollees and number enrolled on TasP and PrEP over time for the high-budget baseline scenario. Each panel shows the number of possible enrollees for each intervention, high-risk susceptibles (*S*_*H*_) for PrEP and chronic infecteds (*C*_*H*_ + *C*_*L*_) for TasP in solid red and blue lines respectively; and the number on each intervention in dashed red and blue lines for PrEP and TasP respectively. The vertical facets define the intervention allocation, the top row (“Optimal”) refers to the DOCP; in the 1-year PrEP case the optimal and only-TasP allocations are the same.

Homogeneous TasP or PrEP have different patterns of effectiveness over time. While TasP’s effectiveness is initially relatively low, it gradually increases in future years. In contrast, PrEP’s effectiveness rises rapidly, but soon after gradually declines; this is observed even when PrEP’s duration is unlimited. Because of its rapid rise in effectiveness, PrEP is almost always superior to TasP in the short term. However, because PrEP effectiveness eventually diminishes while TasP’s effectiveness is still increasing, this superiority is often temporary. This temporary superiority can persist for decades when PrEP is ultimately less effective than TasP (Fig 2, right panel, “1-Year- PrEP” column). PrEP effectiveness diminishes over time because the fraction of high-risk persons that are on PrEP rises rapidly, then begins to decline as new high-risk susceptible begging to enter the model. Fig 3 shows the number of people enrolled into PrEP and TasP and the number of potential targets for those interventions. In the Fig 3 the “Only-PrEP” row shows that the number of high-risk susceptible increases faster than the number of people on PrEP after a PrEP-only program is initiated. Over time, the fraction of the high-risk susceptibles that are protected declines, leading to worse outcomes.

The annual number of persons enrolled into each intervention characterizes the DOCP (Fig 2-left). In these baseline scenarios we find homogenous DOCPs—that is, the DOCP suggests allocation for only one control—in all but one situation. Where the DOCP is homogenous, PrEP duration determines which intervention is optimal. However, in the high budget, “Unlimited-PrEP” situation there is an interesting trade-off. An initial mixed investment in both TasP and PrEP moving to PrEP-only can temper some of diminishing effectiveness of a PrEP-only intervention by increasing the number of treated persons in the period where the effectiveness of PrEP starts to diminish. This is due to the fact that we implicitly model the potential diminishing effectiveness of fixed-budget interventions.

When either PrEP or TasP has a large effect on transmission dynamics, cost-per-enrollee changes, altering the number of people that can be enrolled given a fixed budget. When the PrEP-only control is most effective (Fig 2: “Unlimited PrEP” and “Medium Budget”) the intervention both decreases the number of possible enrollees by enrolling them on PrEP, but also increases the number of possible enrollees by blocking future transmissions from the persons on PrEP, in this case, these effects nearly balance out leading to constant enrollment. However, when TasP is most effective (Fig 2: “1Year PrEP” and “High Budget”) the intervention has the opposite effect, increasing the cost of future enrollment by reducing the intervention’s possible enrollees though both direct enrollment and blocking secondary transmissions leading to fewer people enrolled each year. Thus, if TasP-based intervention is chosen by policy makers, and steady enrollment numbers are desired, the annual budget must be increased yearly to offset the increased cost of enrollment.

### Sensitivity Analysis

**Fig 4:**
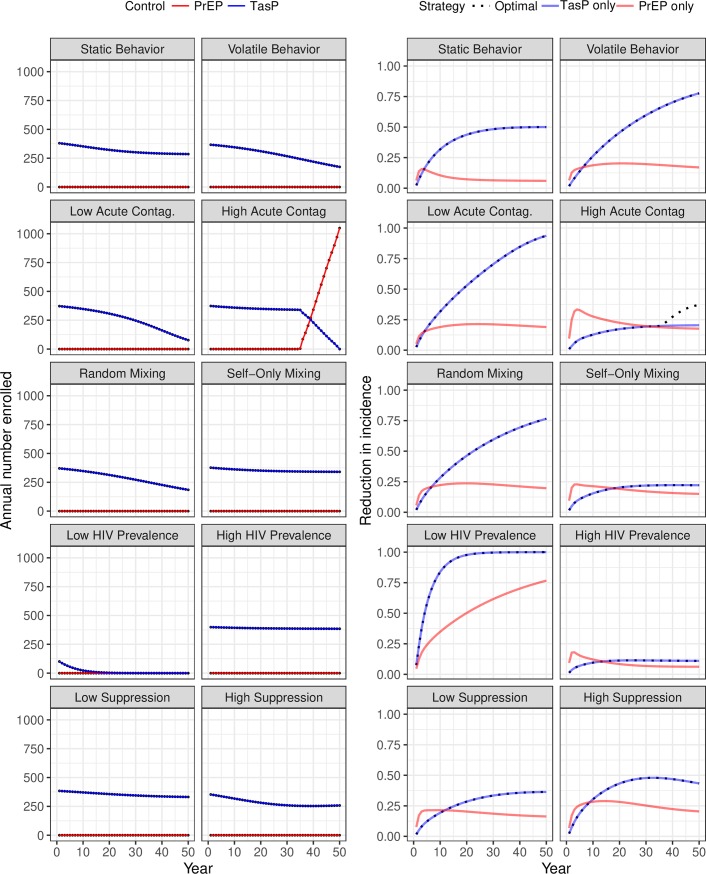
Sensitivity of dynamic optimal control policies and intervention effects to model formulation for the “1-Year PrEP” scenario given an annual budget of 5 million dollars. The optimal number of annual enrollment into PrEP (red) and TasP (blue) interventions is plotted on the left while the multiplicative-scale annual reduction in annual incidence for the optimal (black dots), PrEP-only (blue), and TasP-only (red) interventions is plotted on the right. Each row represents a sensitivity axis where the baseline parameter set, (Fig 2), can be thought of as being between the two extremes of each axis. Parameter sets are described in the materials and methods section.

Where PrEP was only administered for one year, the resulting DOCP was TasP-only over a wide range of scenarios, with only the high acute-stage contagiousness scenario deviating from this pattern (Fig 4). This consistency shows that the qualitative aspect of the DOCP may be robust even when the model is non-identifiable. On the other hand, the quantitative aspects of the DOCP (annual enrollment and annual reduction in incidence) are highly sensitive to the model formulation.

**Fig 5:**
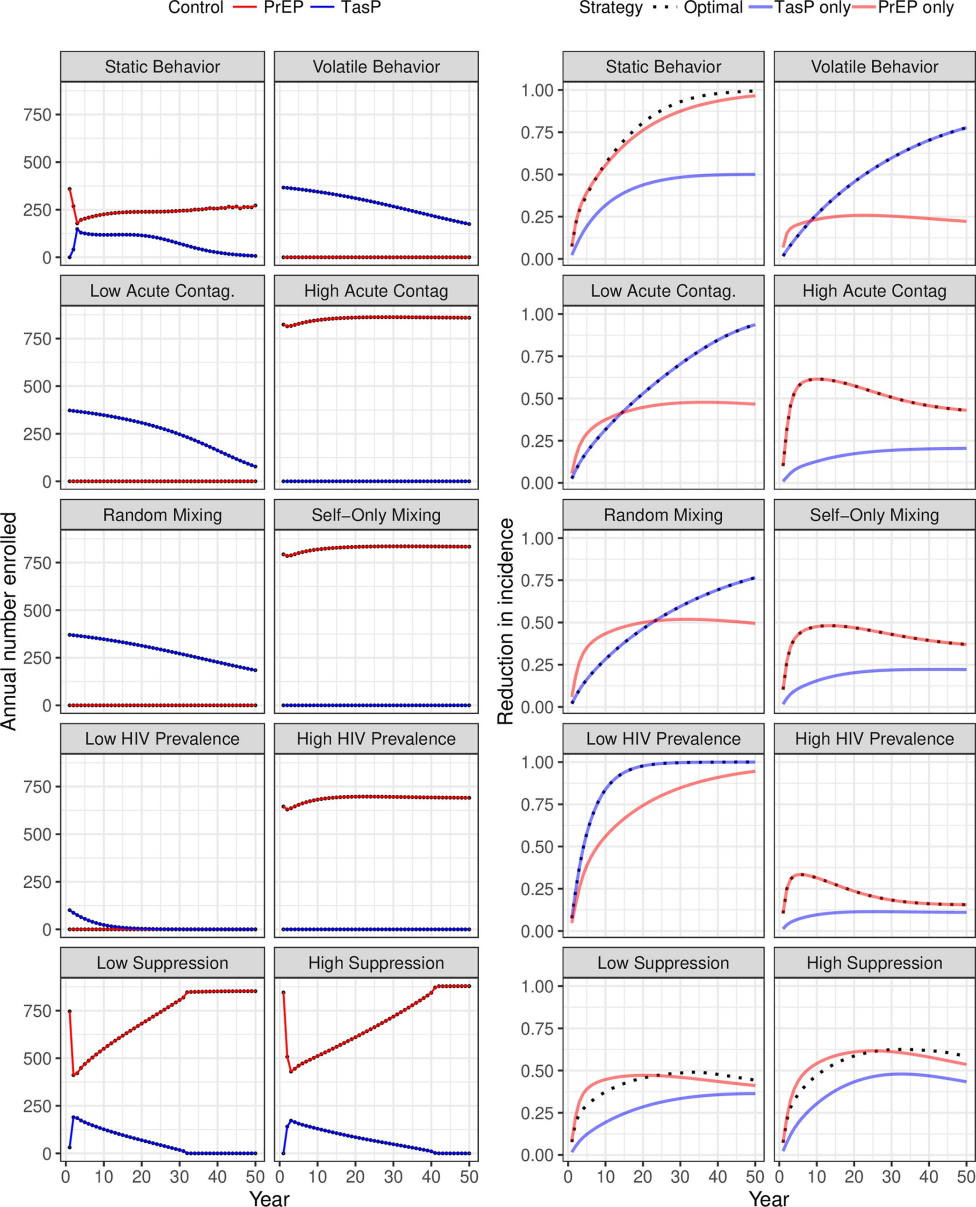
Sensitivity of dynamic optimal control policies and intervention effects to model formulation for the “Unlimited PrEP” scenario given an annual budget of 5 million dollars. The optimal number of annual enrollment into PrEP (red) and TasP (blue) interventions is plotted on the left while the multiplicative-scale annual reduction in annual incidence for the optimal (black dots), PrEP-only (blue), and TasP-only (red) interventions is plotted on the right. Each row represents a sensitivity axis where the baseline parameter set, (Fig 2), can be thought of as being between the two extremes of each axis. Parameter sets are described in the materials and methods section.

In contrast, when PrEP was assumed to be taken for the full duration of a high-risk behavioral episode (Fig 5), we observed the qualitative aspect of the DOCP to be highly sensitive to the model formulation; this relationship holds regardless of the budget (S1, S2, S3 and S4 Figs). The “Static Behavior” panel in Fig 5 illustrates an interesting DOCP that we only observe in this one case. Here the heterogeneous DOCP is unambiguously superior to either of the homogenous controls as it is equal or superior both in the short-term and long-term. This is likely due to the fact that in the static behavior setting, PrEP is assumed to be taken indefinitely due to the assumption of lifelong high-risk behavior. Thus, in this one setting, the dynamics of PrEP and TasP are qualitatively similar because both are assumed to be lifelong. Identifying scenarios and real populations where similar conditions exist may provide new prevention opportunities by simply rearranging the relative resource allocation between prevention modalities rather than increasing resources.

## Discussion

We found that qualitative aspects of the DOCP (being either TasP-only, PrEP-only, or a combination of TasP and PrEP) were largely determined by the duration of PrEP administration. Budget level had no influence qualitatively, though, unsurprisingly, higher budget levels resulted in greater incidence reductions. Although both of these results are not surprising per se, they illustrate that programmatic factors such as duration of PrEP administration can strongly influence the effectiveness of intervention strategies. The importance of this observation is amplified by the fact that DOCPs were found to be qualitatively robust to a wide range of model formulations across the behavioral and biological spectrum. These behavioral and biological factors have been the focus of many studies of the transmission dynamics of HIV and prevention effectiveness; however, we hypothesize that, when addressing the question of how to optimally allocate limited resources, programmatic and cost-related variables may be as or more important as the behavioral characteristics of different populations. Future studies should focus on disentangling these effects in the context of ongoing intervention efforts.

DOCPs were also strongly influenced by the *analytic horizon*, in other words, how far into the future do we consider when we predict the effects of the current intervention. Initially we noticed that in most scenarios the DOCP shifted from homogeneous TasP to heterogeneous PrEP and TasP near the end of the study period regardless of the length of the study period. For example, if optimizing over a 25-year period, the DOCP would shift to PrEP in years 20–25. However, with the same parameterization optimizing over a 50-year period, the shift to PrEP would occur in years 45–50. This effect turned out to be due to the changing analytic horizon. When the algorithm searches for the DOCP over a 50-year window, the horizon in year 1 extends 49 years into the future, however by year 40, the horizon only extends 10 years into the future. That is, by definition, as you approach the end of the study period, the algorithm favors short-term solutions. We addressed this by optimizing over a longer period and discarding the end of the optimization (S5 Fig). It’s tempting to think that this is the universally “correct” solution, but the analytic horizon is another model parameter that needs to be defined *a priori*. For example, if an institution believes that an efficacious and inexpensive vaccine will be released in 10 years, then perhaps current intervention strategies should only be optimized over this 10-year period, a relatively short horizon which would then favor interventions that are more effective in the short-term like PrEP.

Our study comes to a different conclusion than a recent study that argued that the acute-stage transmission rate is not a determinant of the long-run effectiveness of TasP [39]; although the generality of this conclusion is debated [40]. We found that increased acute-stage contagiousness greatly reduced the effectiveness of TasP over a 50-year intervention period. In fact, the preference for PrEP in scenarios with very high acute-stage contagiousness are due to the reduced effectiveness of TasP rather than increased effectiveness of PrEP. This discrepancy is likely due to the fact we assume that the infection is endemic at the start of the intervention. When the endemic risk is fixed as it is in our model, increases in the acute-stage contagiousness must be offset by a reduction in the overall sexual activity level, however these changes nearly balance each other out (Table 3) giving very similar values of the basic reproduction number in the acute-stage contagiousness dimension.

We showed that our algorithms are capable of finding complex DOCPs (involving allocation of multiple intervention resources over time) in a high dimensional parameter space—the main analyses involved optimizing over 150-dimensional space of highly co-dependent parameters. Presently our algorithms are tailored to analyzing this specific model with TasP and PrEP controls, however, we are working to generalize our approach to a more general class of deterministic transmission models and interventions. Given the complexity of these problems the algorithms will require additional basic research and development before they can be thought of as a generalized method.

We believe that modeling interventions for decision support is a local endeavor that requires explicit modeling of actual interventions. Understanding how population and individual-level parameters change the effects of interventions over time is difficult. However, layering on the economic and programmatic factors that are required to fully specify an intervention model makes intuitive analysis a near impossibility. We have demonstrated how methods from control theory can be applied to practical epidemiologic questions focused on intervention design and decisions support.

## Supporting information

S1 Fig Sensitivity of Dynamic optimal control policies and intervention effects to model formulation for the “1-Year PrEP scenario given an annual budget of 1 million dollars. The optimal number of annual enrollment into PrEP and TasP interventions is plotted on the left while the multiplicative-scale annual reduction in incidence is plotted on the right. Parameter sets are described in the materials and methods section.
(TIFF)

S2 Fig Sensitivity of Dynamic optimal control policies and intervention effects to model formulation for the “Unlimited PrEP” scenario given an annual budget of 1 million dollars. The optimal number of annual enrollment into PrEP and TasP interventions is plotted on the left while the multiplicative-scale annual reduction in incidence is plotted on the right. Parameter sets are described in the materials and methods section.
(TIFF)

S3 Fig Sensitivity of Dynamic optimal control policies and intervention effects to model formulation for the “1-Year PrEP” scenario given an annual budget of 3 million dollars. The optimal number of annual enrollment into PrEP and TasP interventions is plotted on the left while the multiplicative-scale annual reduction in incidence is plotted on the right. Parameter sets are described in the materials and methods section.
(TIFF)

S4 Fig Sensitivity of Dynamic optimal control policies and intervention effects to model formulation for the “Unlimited PrEP” scenario given an annual budget of 3 million dollars. The optimal number of annual enrollment into PrEP and TasP interventions is plotted on the left while the multiplicative-scale annual reduction in incidence is plotted on the right. Parameter sets are described in the materials and methods section.
(TIFF)

S5 Fig Burn-out period for Baseline parameter set. This figure shows the full 75-year optimization period for all budget levels in the “1-Year PrEP” scenario. The effect of the intervention horizon on the DOCP is illustrated as the intervention moves closer to the left-hand boundary the horizon gets successively shorter preferring shorter-term solutions.
(TIFF)

S1 File MATLAB code used to compute DOCPs. (M)
