# Correction: Sensitivity of joint contagiousness and susceptibility-based dynamic optimal control strategies for HIV prevention

**DOI:** 10.1371/journal.pone.0212251

**Published:** 2019-02-07

**Authors:** Ingo Bulla, Ian H. Spicknall, Dmitry Gromov, Ethan Obie Romero-Severson

The second author’s name is spelled incorrectly. The correct name is: Ian H. Spicknall. The correct citation is: Bulla I, Spicknall IH, Gromov D, Romero-Severson EO (2018) Sensitivity of joint contagiousness and susceptibility-based dynamic optimal control strategies for HIV prevention. PLoS ONE 13(10): e0204741. https://doi.org/10.1371/journal.pone.0204741
