# Supplementary figures and images for: Sensitivity of joint contagiousness and susceptibility-based dynamic optimal control strategies for HIV prevention
Source: PLoS One. 2018 Oct 18;13(10):e0204741. doi: 10.1371/journal.pone.0204741 (PMC6193630; doi:10.1371/journal.pone.0204741)

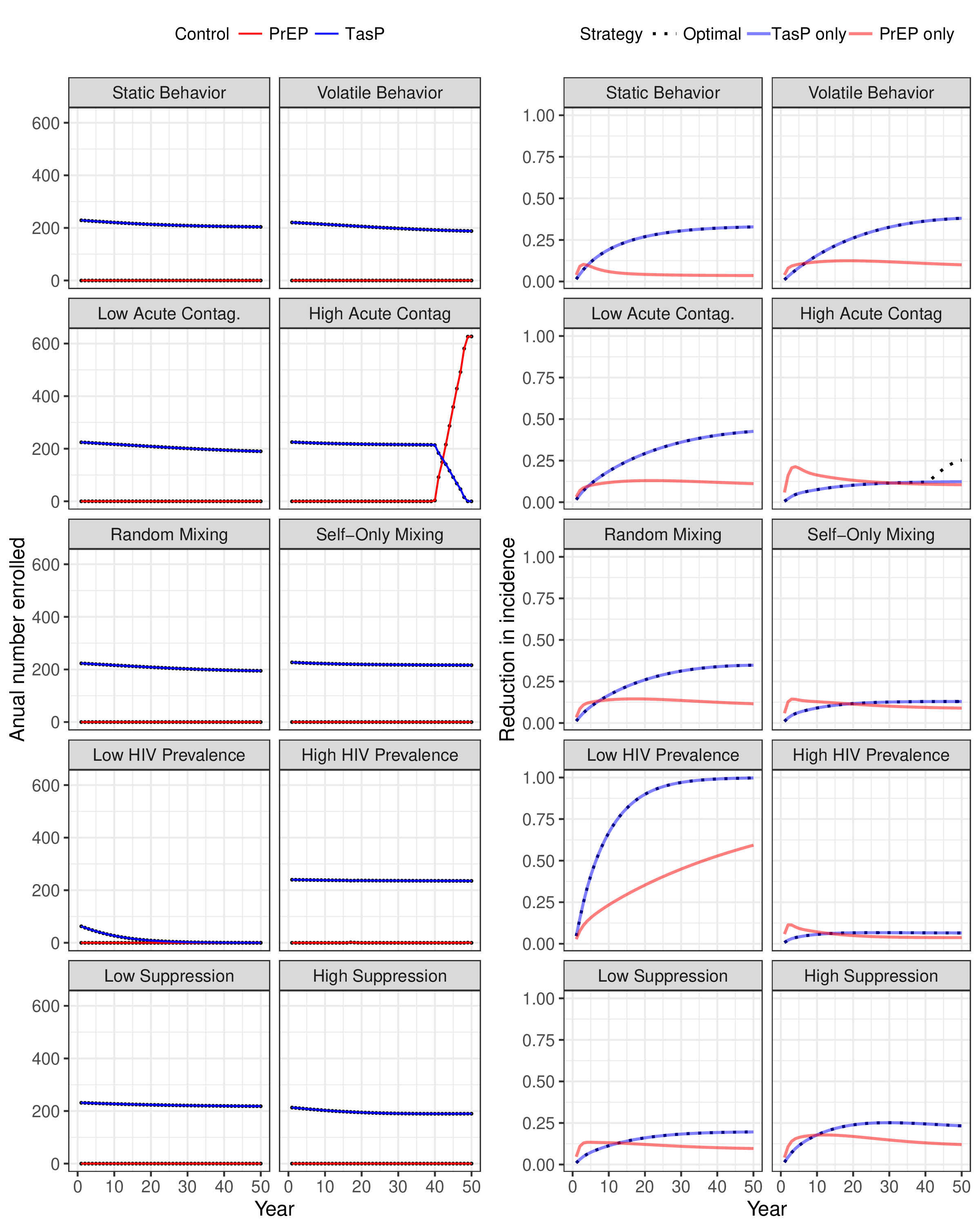

Supplement: S1 Fig — The optimal number of annual enrollment into PrEP and TasP interventions is plotted on the left while the multiplicative-scale annual reduction in incidence is plotted on the right. Parameter sets are described in the materials and methods section. (TIFF) [file pone.0204741.s001.tiff]

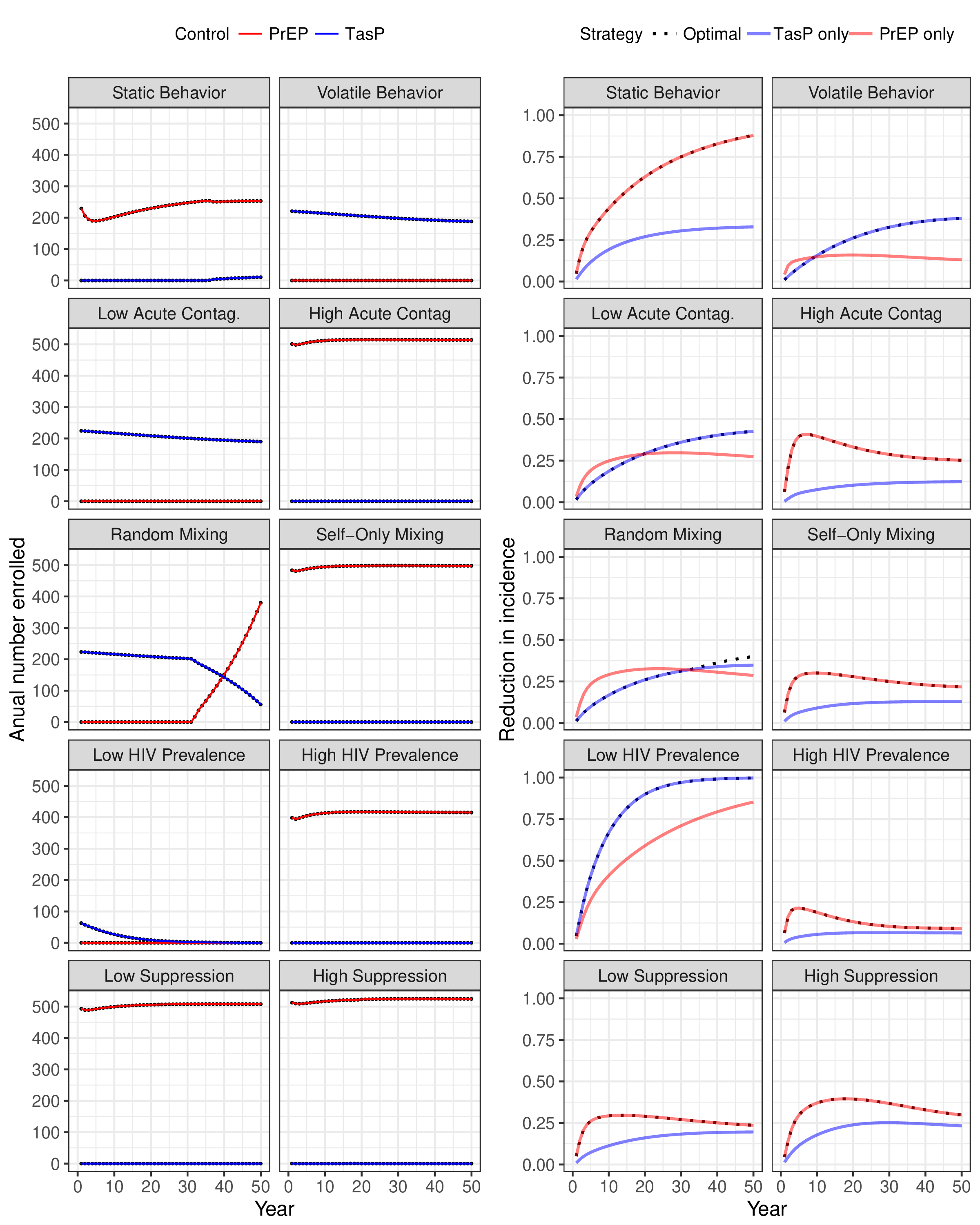

Supplement: S2 Fig — The optimal number of annual enrollment into PrEP and TasP interventions is plotted on the left while the multiplicative-scale annual reduction in incidence is plotted on the right. Parameter sets are described in the materials and methods section. (TIFF) [file pone.0204741.s002.tiff]

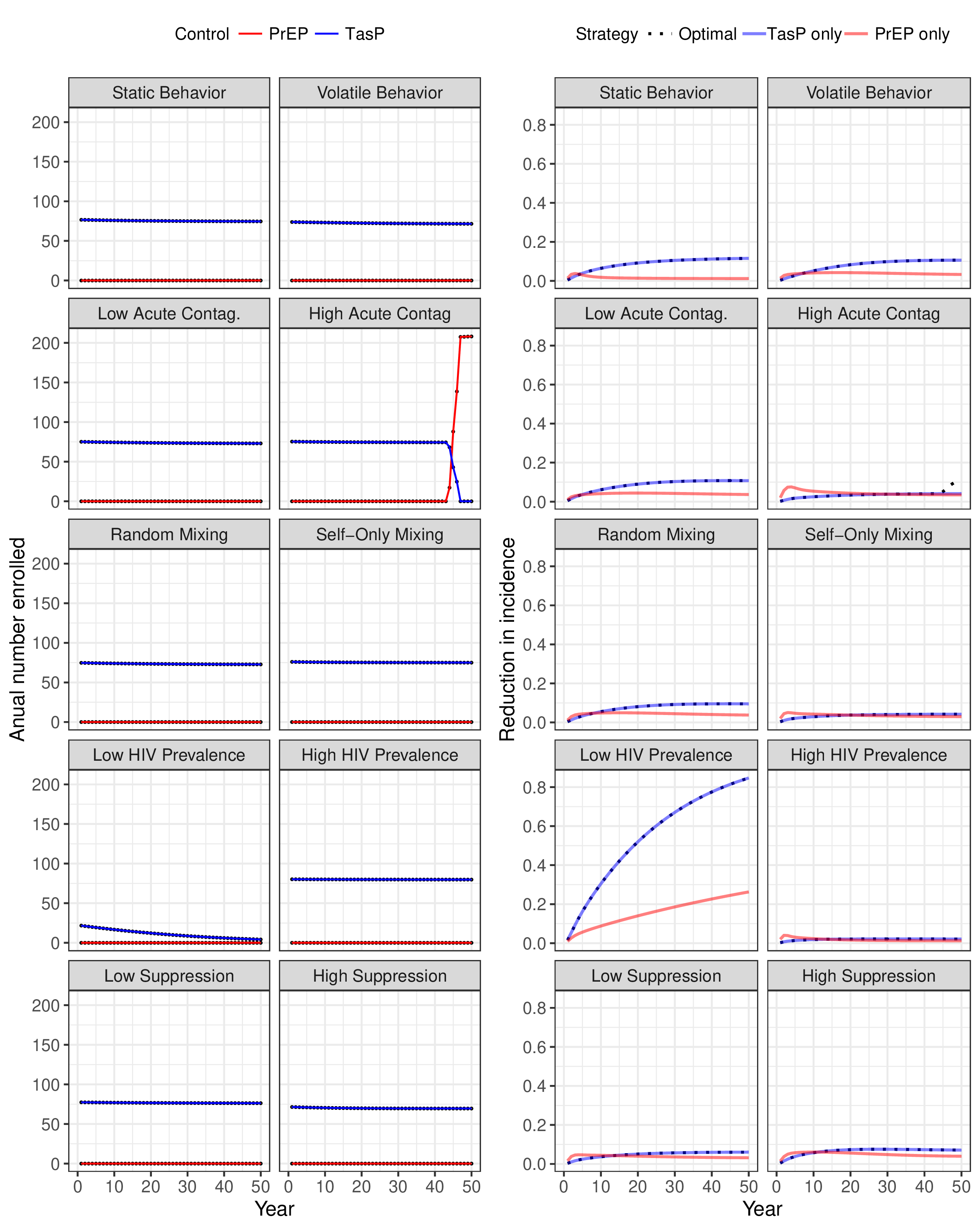

Supplement: S3 Fig — The optimal number of annual enrollment into PrEP and TasP interventions is plotted on the left while the multiplicative-scale annual reduction in incidence is plotted on the right. Parameter sets are described in the materials and methods section. (TIFF) [file pone.0204741.s003.tiff]

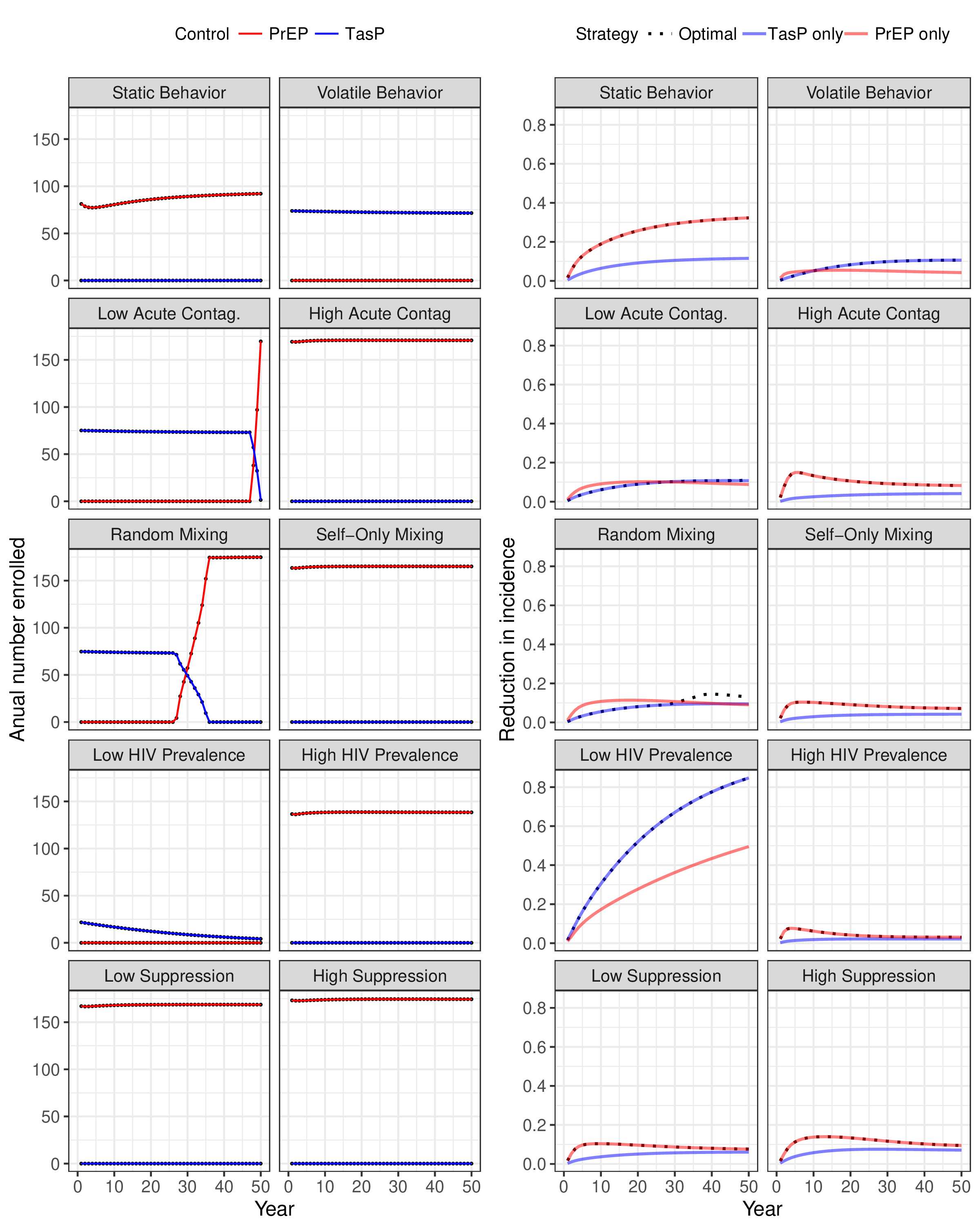

Supplement: S4 Fig — The optimal number of annual enrollment into PrEP and TasP interventions is plotted on the left while the multiplicative-scale annual reduction in incidence is plotted on the right. Parameter sets are described in the materials and methods section. (TIFF) [file pone.0204741.s004.tiff]

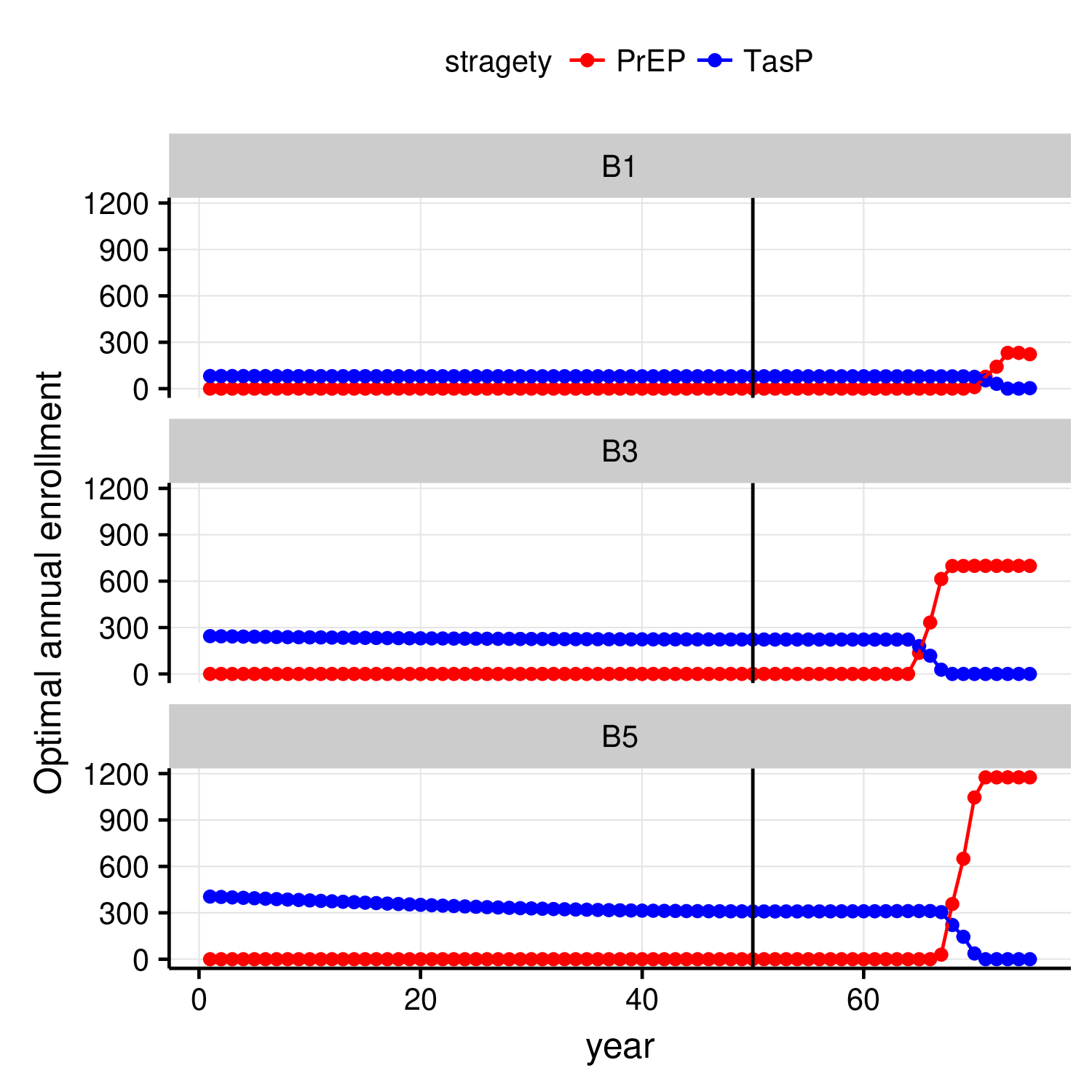

Supplement: S5 Fig — This figure shows the full 75-year optimization period for all budget levels in the “1-Year PrEP” scenario. The effect of the intervention horizon on the DOCP is illustrated as the intervention moves closer to the left-hand boundary the horizon gets successively shorter preferring shorter-term solutions. (TIFF) [file pone.0204741.s005.tiff]
